# The Influence of HLA on HIV-Associated Neurocognitive Impairment in Anhui, China

**DOI:** 10.1371/journal.pone.0032303

**Published:** 2012-05-31

**Authors:** Rachel D. Schrier, Saurabh Gupta, Patricia Riggs, Lucette A. Cysique, Scott Letendre, Hua Jin, Stephen A. Spector, Kumud K. Singh, Tanya Wolfson, Zunyou Wu, Kun Xue Hong, Xin Yu, Chuan Shi, Robert K. Heaton, the HNRC Group

**Affiliations:** 1 Department of Pathology, University of California San Diego, La Jolla, California, United States of America; 2 Department of Psychiatry, University of California San Diego, La Jolla, California, United States of America; 3 Department of Medicine, University of California San Diego, La Jolla, California, United States of America; 4 Department of Pediatrics, University of California San Diego, La Jolla, California, United States of America; 5 Brain Sciences, University of New South Wales, Sydney, Australia; 6 National Center for AIDS/STD Control and Prevention, Beijing, China; 7 Beijing Mental Health Institute, Peking University, Beijing, China; University of Rochester, United States of America

## Abstract

**Background:**

HLA-DR*04 was identified as a predictor of HIV-Associated neurocognitive disorder (HAND), low CD4 T-cell responses to HIV, and low plasma HIV RNA levels in a U.S. cohort. We hypothesized that low CD4 T-cell activation leads to poor immune control of HIV in the CNS, predisposing to HAND, but also provided fewer target (activated CD4 T-cells) for HIV replication. To assess the consistency of these HLA Class II associations in a new cohort and extend analysis to HLA Class I, HLA types, neurocognitive, and virologic status were examined in a cohort of former plasma donors in China.

**Methods:**

178 HIV infected individuals in Anhui China, were HLA typed and underwent neurocognitive evaluations (using locally standardized norms), neuromedical, treatment and virologic assessments at baseline and at 12 months.

**Results:**

HLA DR*04 was associated with a higher rate of baseline neurocognitive impairment (p = 0.04), neurocognitive decline (p = 0.04), and lower levels of HIV RNA in plasma (p = 0.05). HLA Class I alleles (B*27,57,58,A*03,33) that specify a CD8 T-cell response to conserved HIV sequences were neuroprotective, associated with less impairment at baseline (p = 0.037), at month 012 (p = 0.013) and less neurocognitive decline (p = 0.023) in the interval. Consistent with the theory that effective CD8 T-cell responses require CD4 T-cell support, the HLA DR*04 allele reduced the neuroprotective effect of the Class I alleles. The presence of HLA-DR*04 and the Alzheimer associated allele ApoE4 in the same individual had a synergistic negative effect on cognition (p = 0.003).

**Conclusions:**

Despite major background differences between U.S. and Anhui China cohorts, HLA DR*04 predicted neurocognitive impairment and lower plasma HIV RNA levels in both populations. HLA Class I alleles associated with CD8 T-cell control of HIV were associated with protection from HAND, but protection was reduced in the presence of HLA-DR*04.

## Introduction

Differential levels of immune control of HIV and highly variable disease course suggest that host genetics influence HIV disease progression. Since HLA A, B, and DRB1 gene loci specify immune cell recognition and activation, potentially influencing both protective and pathogenic responses, HLA genes are logical genetic candidates. HLA association studies are complicated by the extensive diversity at the 3 main loci (A, B, DRB1) and the requirement for large sample sizes. Despite these obstacles, independent studies have now linked specific HLA types with high or low risk for HIV seroconversion, immunologic control, and disease progression [1], [2], [3], [4], [5], [6], [7], [8]. Findings from a number of groups agree that certain HLA A and B alleles (HLA B*27 B*57, 58, in US and Western European cohorts) present conserved regions of HIV gag and nef to T-cells [1], [9], [10], [11], [12]. The hypothesis is that individuals with these alleles have effective anti-HIV CD8 T-cells that maintain immune control of HIV because virus escape mutations in these conserved regions result in significant loss of replicative fitness [10], [13], [14]. Therefore the CD8 T-cells in these individuals tend to remain effective and if escape mutants are viable, they grow slowly. In either case, individuals with such alleles tend to have a slower rate of HIV disease progression. However, it is not yet clear whether there are specific alleles that increase or decrease risk of HIV associated neurocognitive disorder (HAND) or whether alleles that are linked to slow disease progression in general also protect from HAND.

Mild to moderate neuropsychological impairment can be detected in 30–40% of HIV infected cohorts, and affects an individual’s ability to work, day to day functioning, and quality of life [15], [16], [17] [18]. Antiretroviral treatment (ART), while significantly reducing incidence of HIV encephalitis, plasma HIV RNA, and reconstituting CD4 numbers and resistance to opportunistic infections, has had a less dramatic impact on chronic HAND, increasing the relative visibility of moderate cognitive impairment in otherwise well managed cohorts [17]. As it becomes feasible to target CNS infection therapeutically, there is increasing interest in identifying genetic phenotypes that either predispose or protect the host from HAND. In an earlier cohort of 191 HIV-infected, HLA typed persons in the United States [19], HLA DR alleles that presented relatively few HIV peptides (especially HLA DR*04) were associated with a higher risk of HAND, but also, unexpectedly, low plasma HIV RNA levels. In contrast, those individuals with HLA DR types with broad peptide recognition had a lower risk of HAND, but comparatively higher HIV RNA levels [4]. Our explanation for the differences in HIV RNA levels was that individuals with broad response alleles had more activated CD4 T-cells to host HIV replication, while those with low CD4 responses (and activation) had fewer permissive host cells and consequently less HIV replication. The higher risk of HAND in the low CD4 response subgroup could be due to a lack of CD4 help for HIV specific CD8 T-cells, whose role is to migrate into the CNS and destroy HIV infected cells. Lack of CD4 support for CD8 T-cells [20] would permit spread of HIV and associated inflammation, leading to greater risk of neurocognitive impairment.

Recent studies from other groups are relevant to our initial report. HLA DR*04, which was linked to low (and narrow) CD4 T-cell responses to HIV is also reportedly associated with low CD4 T-cell responses to mitogens in Autism Spectrum Disorder [21], [22]. In the HIV field, specific HLA Class I (A and B) alleles that encode for protective CD8 recognition have been identified. HLA Class I alleles (B*27, B*57, B*58) that present conserved regions (mostly gag and nef) of HIV appear to protect against HIV disease progression and are at higher frequencies in long term non progressor and virus controller groups [1], [10], [12], [23],[24],[25].

A robust method of testing genetic hypotheses is to re-examine the allele associations in a new, non-overlapping cohort. Our research group’s study in China presented an opportunity to validate our previous findings concerning HLA and HAND and extend our investigation in a new and ethnically different population. Several studies in China have reported on associations between HLA genes, HIV seroconversion and progression to an AIDS diagnosis, as well as T-cell recognition, providing important information on local allele frequencies for updating hypotheses. Based on our previous results and (other’s) findings for Class I alleles, we proposed to assess whether the HLA Class II allele previously identified, HLA DR*04, was associated with neurocognitive impairment, decline, and low HIV RNA levels. We also asked whether Class I alleles that present conserved peptides of HIV and are associated with slow disease progression (in the US and China) are also associated with protection from neurocognitive impairment. In addition, because a previous study of genetic variants associated with neurocognitive impairment in this cohort identified an association with ApoE4, the role of ApoE4 in mediating the HLA effects was also examined [26].

## Methods

### Participants

The grant that supported this study: “The Neurobehavioral Effects of HIV infection and Host Genetics in China” was approved by the University of California San Diego Institutional Review Board (IRB), and the IRBs of the National Center for AIDS/STD Control and Prevention, Beijing, China and Beijing Mental Health Institute, Peking University. Data collection, confidentiality, and genetic analyses were as proposed in the initial IRB approved grant and described in the consent forms signed by all participants. Located in rural Anhui, China, the cohort was composed of former plasma donors. Neuropsychological norms were established by assessing local HIV- individuals, matched with the HIV+, for age, education, gender and the infection risk factor of being a former plasma donor. Neuropsychological evaluations were translated into Mandarin. Chinese psychiatric personnel were trained by our San Diego team. Epidemiologic, treatment, clinical, and neurobehavioral data were updated at yearly intervals [15]–[16]. Participant recruitment and baseline findings have been published Heaton et al. [15]. Briefly, at baseline 203 HIV+ and 198 HIV-participants, farmers and laborers in the rural area of Anhui province, were enrolled into the study at a local hospital in Fuyang City. All available HIV+ participants were re-assessed at 12 months (N = 192), a 5.4% (11/203) attrition rate. Only HIV+ participants were HLA typed. The initial number of HLA typed individuals was 187, falling to 178 at the 12 month assessment. Of the 178 HLA typed individuals, 96 (53%) met CDC 1993 criteria for AIDS at baseline and were on ART. Of those 96, 47 (49%) were HIV plasma RNA undetectable (<50 copies/ml). The most common ART regimen was D4T/ddI/NVP (62%) and the second most common was D4T/3TC/NVP (35%). After 12 months, 19 additional HIV+participants had progressed to AIDS, and 60.9% of the total group was receiving ART. Sixty-one percent of the cohort was male, with an average of 5.5 years of education. Fifty-one percent of the 178 were HBV surface antibody positive and 94% were HCV antibody positive (in serum). Self reported HIV infection duration was estimated at 154 months (Standard deviation = 42).

By design at 12 months post-baseline, half of the baseline HIV- participants (N = 101) were re-assessed in order to develop normative standards for neuropsychological change. The 101 HIV-participants had been randomly selected in order to compose a sample representative of the total baseline group with respect to demographic and baseline neuropsychological characteristics [27].

### Procedure

Participants underwent comprehensive neurocognitive and neuromedical evaluations, and a structured psychiatric examination. Examinations (baseline and follow-up) were performed by Chinese psychiatric staff trained and certified in the standardized testing procedures by the U.S. research team (RKH). Details of the neuropsychology battery selection and adaptation for use in China are provided in Cysique et al. [16], and Heaton et al. [15]. Neurocognitive impairment status was defined by the global deficit score (GDS), that represents a summary score for multiple cognitive domains (Verbal Fluency, Attention/Working Memory, Speed of Information Processing, Learning, Delayed Recall, Executive Function, and Motor Function) and ranges from 0–5, with values above 0.5 representing clinically significant impairment. The GDS can be used as both a continuous and dichotomous variable [18]. Based upon the data of the 101 HIV- controls who received 12 month follow-up examinations, separate norms were developed to control for practice effect and to classify significant change on the neuropsychological test battery [27], [28] Participants also completed the Beck Depression Inventory-II (BDI-II [29]).

### HLA Typing and Laboratory Tests

HLA typing and most laboratory tests were performed at the China CDC in Beijing. DNA was extracted from whole blood using Qiagene kits. Two digit low resolution DNA typing kits, purchased from Texas Biogene, were used as directed. Accuracy of database information (including HLA typing and laboratory tests) was assessed by requests for repeat sending of random samples. ApoE genotypes were detected in Dr Spector’s laboratory using DNA extracted from dried blood spots using real-time PCR (LightCycler, Roche) as described previously [26].

### Data Analysis

Continuous variables were compared using two-tailed t-tests (Table 1) or two-sided Wilkinson non-parametric tests (Tables 2, 3, 4, 5). Non-normally distributed variables in Table 1 (GDS, HIV RNA) were square-root transformed or log transformed, respectively and variances for the compared groups were similar (Levine test). Chi-square (groups >30) or Fisher Exact test were used for dichotomous variables (identified by an asterisk in tables). Our HLA analyses were initially performed by separating individuals into neurocognitively impaired and unimpaired groups and calculating allele frequencies for HLA A, B, and DR loci, and second, by selecting groups with and without specific alleles of interest and comparing average values of clinical measures. Because of the extensive polymorphism at the HLA locus, the issue of multiple comparisons must be addressed, either by a two-stage analysis or a gross correction of the p value (generally considered to be overly conservative). Given that specific predictions were formulated (based on a previous study) prior to data collection, the analyses shown in the data tables represented the second stage of the analysis, so that no additional adjustments were necessary. We hypothesized that HLA DR*04 would be at a higher frequency in the group with neurocognitive impairment since it was the strongest predictor in previous studies. New observations with (uncorrected) p values are identified as such in the results, but not shown in tables. All p-values shown are two-sided.

**Table 1:** Individuals with HLA DR*04 tend to be more impaired but also have lower HIV RNA.

Measure	Interval	DR*04+	DR*04−	p value
N		42 (22%)	136	**(t-test)**
GDS	Baseline	0.69	0.57	**0.04**
GDS	12 mo	0.74	0.62	**0.07**
%ART	Baseline	60%	52%	0.48*
CD4	Baseline	369	364	**0.44**
CD8	Baseline	924	882	**0.3**
CD4	Nadir	267	276	**0.47**
HRNAlog	Baseline	2.98	3.39	**0.08**
HRNAlog	12 mo	2.86	3.36	**0.04**
				*FET

Values (other than sample N) in tables represent averages of the indicated variables. BL is baseline score and 012 is twelve month evaluation point. GDS is ‘global deficit score’ and neurocognitive impairment is defined as ≥0.5. GDS values were square root transformed. Percent ART is percentage of group on at least 2 anti-retroviral medications. CD4 and CD8 value are absolute cells/microliter. HRNA log is log value of HIV RNA copies/ml with a lower limit of detection of 50 copies. All statistical tests are two tailed, values <.05 are underlined and an asterisk denotes Fisher Exact Test.

**Table 2:** Individuals who are DR*04+ and have detectable HIV RNA have a greater risk of neurocognitive decline.

Measure	Interval	DR*04+	DR*04−	DR*04+	DR*04−	RNA+DR*04+	DR*04+RNA+	DR*04+
		RNA+	RNA+	RNA−	RNA−	vs DR*04−	vs RNA−	RNA+
N		22	87	20	49	p =	p =	vs others
NC Decline	BL-012	45%	26%	20%	24%	.06*	.05*	.04*
%ART	12 mo	38%	44%	85%	90%	0.45	0.001	
CD4	Nadir	268	285	266	230	0.33	0.49	
HRNAlog	12 mo	3.91	4.3	1.69	1.69	.04*		

Values (other than sample N) in tables represent averages of the indicated variables. BL is baseline score and 012 is twelve-month evaluation point. “Decline” is significant neurocognitive decline between BL and 012 (see methods). Percent ART is percentage of group on at least 2 anti-retroviral medications. CD4 values are absolute cells/microliter. HRNA log is log value of HIV RNA copies/ml with a lower limit of detection of 50 copies. All statistical tests are two tailed, values <.05 are underlined. A p value with an asterisk denotes Fisher’s Exact Test and others were derived using Wilcoxen non-parametric test.

**Table 3:** HLA Class I alleles that specifiy recognition of conserved HIV sequences protect from neurocognitive impairment and decline.

	Interval	At least one protective Class I allele: (A*03,*33,B*27,*57,*58)	No protective Class I allele	p value FET*, Wilkinson
N		47	131	
% NC Decline	BL-12 mo	15% (7)	31% (42)	.024*
GDS	BL	0.35	0.53	0.014
GDS	12 mo	0.4	0.61	0.008
%On ART	12 mo	60%	63%	0.77*
CD4	Nadir	249	271	0.57
HIV RNA log	12 mo	3.42	3.19	0.323
%RNA<50	12 mo	28%	36%	.55*
%DR*04		21%	23%	.87*

Values (other than sample N) in tables represent averages of the indicated variables. BL is baseline score and 012 is twelve month evaluation point. “Decline” is significant neurocognitive decline between BL and 012 (see methods). GDS is ‘global deficit score’ and neurocognitive impairment is defined as ≥0.5. Percent ART is percentage of group on at least 2 anti-retroviral medications. CD4 and CD8 value are absolute cells/microliter. HRNA log is log value of HIV RNA copies/ml with a lower limit of detection of 50 copies. All statistical tests are two tailed, values <.05 are underlined. A p value with an asterisk denotes Fisher’s Exact Test and others were derived using Wilcoxen non-parametric test.

**Table 4:** The low CD4 response allele, HLA DR*04, limits the efficacy of protective HLA Class I alleles.

Measure	Interval	PCI+	PCI+	PCI−	PCI−	PCI+DR*04-vs
		DR*04−	DR*04+	DR*04−	DR*04+	PCI-DR*04+
N		36	11	100	31	36 vs 31
%NC Decline	BL-12 mo	11%	27%	31%	35%	0.021*
GDS.	Baseline	0.32	0.45	0.53	0.7	0.003
GDS	12 mo	0.35	0.58	0.61	0.75	0.003
CD4	Nadir	245	259	271	272	0.73
HIV RNA	12 mo	3.43	3.38	3.19	2.67	0.032
%on ART	12 mo	61%	64%	59%	68%	0.68*

Values (other than sample N) in tables represent averages of the indicated variables. BL is baseline score and 012 is twelve-month evaluation point. “NC Decline” is significant neurocognitive decline between BL and 012 (see methods). GDS is ‘global deficit score’ and neurocognitive impairment is defined as ≥0.5. Percent ART is percentage of group on at least 2 anti-retroviral medications. CD4 and CD8 value are absolute cells/microliter. HRNA log is log value of HIV RNA copies/ml with a lower limit of detection of 50 copies. All statistical tests are two tailed, values <.05 are underlined. A p value with an asterisk denotes Fisher’s Exact Test and others were derived using Wilcoxen non-parametric test.

**Table 5:** HLA DR*04 and ApoE4 have a synergistically negative effect on cognition.

Measure	Interval			E4+vs			E4-DR*04-vs
		E4+DR*04−	E4-DR*04+	DR*04+	E4-DR*04−	E4+DR*04+	E4+DR*04+
N		33	33	p =	103	9	p =
Edu	(yrs)	5.2	5.7	0.05	5.8	5.1	0.52
%ART	BL	45%	39%	0.80*	55%	67%	0.73*
RNAlog	12 mo	3.74	2.86	0.07	3.22	2.85	0.45
GDS	BL	0.74	0.55	0.91	0.42	0.87	0.02
GDS	12 mo	0.75	0.63	0.32	0.51	0.9	0.07

Values (other than sample N) in tables represent averages of the indicated variables. BL is baseline score and 012 is twelve month evaluation point. Edu is years of education. GDS is ‘global deficit score’ and neurocognitive impairment is defined as ≥0.5. Percent ART is percentage of group on at least 2 anti-retroviral medications. CD4 and CD8 value are absolute cells/microliter. HRNA log is log value of HIV RNA copies/ml with a lower limit of detection of 50 copies. All statistical tests are two tailed and values <.05 are underlined. A p value with an asterisk denotes Fisher’s Exact Test and others were derived using Wilcoxen non-parametric test.

To identify individuals who experienced overall neurocognitive change between baseline and follow-up, a statistical methodology based on the multivariate regression change score approach was used [30], Cysique et al (2010). The regression based change score accounts for practice effect, regression towards the mean and other factors that may influence normal test-retest variability in neurologically stable people (e.g., test-retest interval, demographics, and overall baseline neurocognitive competence [30]. To define neuropsychologic decline in the HIV+ sample, 101 demographically comparable HIV- individuals from the baseline assessment were used as a reference sample to develop normative regression formulas. The final regression formulas were then applied to the HIV+ sample providing a z-score for each of 17 neuropsychologic variables, which reflects the degree and direction of deviation form the predicted follow-up score. These z-scores reflect how well or poorly the person performed at follow-up, relative to normal expectation for someone with same baseline performance and relevant demographic characteristics (e.g. age). The z-scores were then summed to provide a *summary regression change score* (sRCS). A 90% confidence interval was determined on the sRCS to define “no change” on the test battery. That is, the cut-off for the top 5% of the sRCS distribution of the HIV- controls defined the “improved” range and the cut-off for the bottom 5% defined the “decliners” range. This was applied to the HIV+ sample. The percentage of HIV+ “decliners” and “non-decliners” (as defined by the sRCS) in genetically defined groups were then compared using Chi-square or Fisher Exact Test. The GDS T-scores at follow-up were corrected for the HIV- sample median practice effect [15], [16].

## Results

### HLA DR*04 is Associated with Neurocognitive Impairment

Our first analysis addressed whether the HLA DR alleles previously reported to be associated with low CD4 T-cell responses and neurocognitive impairment in the US were similarly associated with neurocognitive impairment in Anhui China. To avoid conclusions biased by a single analytic approach, data were analyzed both cross-sectionally and longitudinally. At baseline (BL) 64 of178 (36%) of individuals in the HIV+ HLA typed group were considered “impaired” with a global deficit score (GDS) greater than 0.5. The frequency of HLA DR*04 (22% for the parent dataset) was significantly higher in the impaired Anhui subjects (31%) compared to the non-impaired group (17%, p = 0.039 by Chi Square).

To further examine the HLA DR*04 associations, those individuals with one or more copies of HLA DR*04 (N = 42 of 178) were compared with those without HLA DR*04 (N = 136 of 178), and clinical measures compared. Group averages are presented in Table 1 with p-values determined by two-tailed t-test. Although the HLA DR*04+ and DR*04− groups were similar in terms of ART, CD4 levels, and nadir CD4, those with HLA DR*04+ were more impaired at both baseline and at 12 months, compared to those without HLA DR*04. HIV RNA levels were lower in those with HLA DR*04 (both at baseline and 12 months) compared to those without. Individuals with DR*04 were also more depressed (higher BDI) at baseline but not at 12 months. Other alleles higher in the baseline impaired group, but not predicted by our previous studies were: HLA B*13 (25% versus 2.5%, p = 0.005) and HLA B*15 (32% versus 5%, p = 0.011) by Fisher’s exact test. HLA B*15 has been identified as a risk factor for general HIV disease progression in other studies in China, but no mechanism has been described [3], [6].

### HLA DR*04 is Associated with Neurocognitive Decline in Participants with Detectable HIV RNA

Since the cross sectional correlations between impairment and HLA alleles reported in Table 1 could represent HIV independent mechanisms (such as HLA DR*04′s reported association with ASD [21]), we also examined neurocognitive decline as a function of HIV RNA levels. Comparison of neurocognitive decline during one year of uncontrolled (compared to controlled) HIV infection should highlight an HIV associated mechanism. Uncontrolled HIV disease was defined as detectable HIV RNA (≥50 copies/ml, with or without ART) during the 12 month period. Forty-nine of 178 (27%) HLA typed individuals qualified as significant neurocognitive “decliners” (based on the 95% summary “change score” cutoff). Neurocognitive decliner and non-decliner groups were stratified according to presence or absence of detectable plasma HIV RNA and HLA A, B, and DR allele frequencies calculated for all 4 groups.

The percent decline and clinical averages for 4 groups (+/−DR*04, +/− HIV RNA) are shown in Table 2. The only (HLA A, B, or DR) allele associated with significant neurocognitive decline in the HIV RNA detectable group was HLA DR*04. Neurocognitive decline was highest (45%) for HLA DR*04 positive individuals with detectable HIV RNA, compared to or those with HLA DR*04, HIV RNA suppressed (20% decline, p = 0.04,) or those without HLA DR*04 and HIV RNA detectable (26% decline, p = 0.06). HIV RNA levels for HLA DR*04 positive RNA detectable group remained lower than the HLA DR*04 negative RNA detectable group, suggesting that the lower level of HIV RNA in the HLA DR*04+ group in Table 1 was not due to a higher frequency of RNA undetectable individuals. Neither HLA B*13 or B*15, which were associated with baseline impairment, were linked to neurocognitive decline. HLA B*46, which has been identified as a risk factor for HIV seroconversion in China [2], however, tended to be more common in the decliner group p = 0.06. HLA B*58, associated with slower HIV progression [14] was rare in this cohort, so that the over-representation in the non decliner group was not statistically significant (p = 0.09).

### Presence of HLA Class I Protective Alleles is Associated with Less Neurocognitive Impairment and Decline

A second major goal of the study was to determine whether HLA Class I alleles (HLA A,B) associated with slower HIV disease progression in other research studies can protect from neurocognitive decline. The protective alleles identified in US and Europe: B*27, B*57, B*58 are, rare in Asia (7, 5, and 6 individuals, respectively in our cohort of 178). However, in the interest of identifying conserved HIV viral epitopes (and the alleles that present them) for local vaccine design, studies in China have systematically analyzed HLA restricted CD8 T-cell recognition. Results validated the identification of B*27, B*57, and B*58, but additional alleles: HLA A*03 and A*33 were also newly identified as recognizing conserved HIV gag sequences [7], [31].

As noted above, in the current study, HLA B*58 was more common among HIV infected participants with stable neurocognitive status. HLA B*27 and 57, the most protective in US studies, were over represented in the non-decliner subset (7 of 7 for B*27), but the frequencies of these 3 alleles were too low in China for analysis individually. Since the question posed was whether the presence of any (rather than one specific) HLA Class I allele (that presented conserved HIV sequences) was associated with protection from neurocognitive impairment and decline, we performed a collective analysis for both the newly identified (in China) and published protective alleles, asking whether the presence of any one or more preserved cognition (i,e, no impairment or decline) compared to persons without. This selection identified 47 of 178 (26%) had one or more copies of: HLA A*03, A*33, B*27, B*57 or B*58 alleles.

Neurocognitive impairment status and decline rates were compared for those with and without the five “protective” Class I alleles and results are shown in Table 3. Percent neurocognitive decline was significantly higher in the group without a protective HLA Class I allele (p = 0.024). In addition, the mean impairment scores (mean GDS at baseline) of individuals with protective Class I (PCI) alleles were significantly lower (p = 0.014), remaining in the normal range (GDS<0.5) over the year of follow-up, while the group without a PCI allele were on average, impaired (GDS ≥.05) at baseline and declined more dramatically. The frequency of ART and presence of the HLA DR*04 allele were comparable in the PCI versus no PCI patient groups. Nadir CD4 and percent HIV RNA undetectable tended to be lower, and level of viremia slightly higher in the PCI group. Ten of the 47 individuals had 2 or more PCI alleles and none showed neurocognitive decline over the year of follow-up (data not shown).

### Presence of HLA DR*04 Diminishes the Protective Effect of Class I Alleles on Neurocognitive Impairment and Decline, But Remains Associated with Lower Plasma Viremia

Effective HLA Class I restricted CD8+ T-cell cytotoxic activity generally requires ongoing support from HLA Class II restricted CD4+ T-cells [20]. This would predict that low CD4 T-cell activation, such as that associated with HLA DR*04, could limit effective cytotoxic activity of PCI CD8+ T-cells against HIV infected cells. To determine if the presence of HLA DR*04 reduced the protective neurocognitive effect of the PCI alleles, the PCI positive and negative groups were subdivided according to presence or absence of HLA DR*04. The 4 groups are shown in Table 4, with a statistical comparison of the 2 extremes (PCI+ DR*04− and PCI- HLA DR*04+). In addition to the lower percent decline, the group with protective Class I alleles tended to decline within the normal range, while the group without protective alleles declined across the impairment cut off and within the impaired range. Considering the mean neurocognitive impairment scores at baseline and follow-up as well as the frequency of neurocognitive decline, the trends suggest that presence of HLA DR*04 reduces the protective effect of the PC1 alleles on cognition. This effect is independent of CD4 and CD8 counts (data not shown), as well as CD4 nadir. Consistent with previous analyses, HLA DR*04 remained associated with lower HIV RNA levels.

### Synergistic Consequences of ApoE4 and HLA DR*04

Allele frequencies for other loci were also analyzed as part of this study. While most of the findings regarding non-HLA loci are published separately [26], we did investigate potential interactions between HLA alleles and non-HLA alleles that influenced cognition in the Anhui cohort. The most intriguing observation related to the ApoE4 allele, which has been associated with Alzheimer’s disease occurrence and severity [32], [33]. Similar to HLA DR*04, ApoE4 was present in 22% of the cohort and associated with baseline neurocognitive impairment. Of note, both alleles are individually associated with syndromes that impact cognition (autism and Alzheimer’s). However, in contrast to HLA DR*04 where the association with neurocognitive decline was observed in the HIV RNA detectable group, ApoE4 was most strongly associated with neurocognitive decline in the antiretroviral treated, HIV RNA <50 group, suggesting different mechanisms of action for the two alleles (data not shown). To determine whether there was an additive or synergistic effect on cognition between HLA DR*04 and ApoE4 when both were present in the same individual, the mean neurocognitive scores of the 4 subgroups for this analysis (ApoE4 only, HLA DR*04 only, Neither, Both) are compared in Table 5, with p values shown below each pair. As the two gene loci are on different chromosomes, (ApoE on 19 and HLA on 6), the two sorted independently. Individuals with either allele alone had mean GDS scores above the impairment cut-off (0.5), but means of individuals with ApoE4 tended to be higher than those with HLA-DR*04. Comparison of degree of impairment of those with both HLA DR*04 and ApoE4 and those with neither allele, revealed significantly greater impairment in the group with both alleles, for both average impairment score at BL and 12 months and percent of those impaired. Specifically, there was a 78% impairment rate among the 9 subjects with both compared to a 34% impairment rate among the other 169 subjects. The interaction was synergistic since there is a statistically significant association between having “both ApoE4 and HLA-DR4” vs. “either one alone or neither” (p = 0.01, two-sided Fisher’s Exact test). ART, CD4 nadirs and levels and CD8 levels were similar for all groups (not shown). Consistent with other group comparisons, plasma HIV RNA levels were lower for individuals with HLA DR*04.

## Discussion

HAND is a chronic aspect of HIV disease that appears to be initiated by HIV and facilitated by immunosuppression. In that sense, protection from neuropsychological impairment would be predicted to correlate with immune and genetic markers (such as HLA*B27, B57, and B58) that are associated with long-term control of HIV viremia and low rate of CD4 depletion. Since not all HIV infected individuals develop HAND (typical estimates are around 40%), genetic associations could identify those at greatest risk for HAND for preventive treatment, as well as contributing to our understanding of pathogenic mechanisms. The findings presented here confirm and extend previous observations [4] that HLA alleles coding for a low CD4 T-cell response (specifically HLA DR*04) are associated with HAND and neuropsychological decline. Decline was measured as change over one year, a relatively short period in the course of HIV disease. However, a higher rate of neuropsychological decline was detected in participants with detectable HIV RNA who had the DR*04 allele (45%), compared to those without the DR*04 allele (25%), those with DR*04 who had detectable HIV viremia (20%), or those without DR*04 and plasma HIV RNA suppressed (24%).

These findings also suggest that despite a relatively short interval (1 year), clinically meaningful changes can be detected and linked to host genetic factors.

The association of HLA DR*04 with low immune responses has been observed in other contexts. It has been shown that T-cells from autistic (HIV-) individuals with HLA DR*04+ proliferate poorly to mitogens [21]. The major protective role played by CD4 T-cells in HIV disease is thought to be their helper function for CD8 T-cells. Without sustained CD4 help, CD8 T-cell cytotoxic activity is transient, even in the presence of chronic viremia [20]. This suggests that poor CD4 support could lead to failure of CD8 cytotoxic activity towards HIV infected cells, even for those individuals with HLA Class I alleles coding for recognition of conserved regions of the virus. Low CD4 activity also generally leads to lower antibody responses.

Although HLA DR*04 was associated with HIV related neurocognitive impairment, it was also significantly related to lower plasma HIV RNA levels (compared to those without DR*04). This is counterintuitive since most symptoms of HIV infection as well as neurocognitive decline overall correlate positively with plasma viremia. However, the association between HLA DR*04 and low plasma viremia was detected previously (in conjunction with low T-cell proliferative responses to HIV), and appears consistently in the data shown in this report. Since HIV replicates best in activated CD4 T-cells, the most straightforward explanation for the HLA DR*04 association with lower plasma HIV RNA levels is that less T-cell activation and proliferation leads to less HIV replication.

Identifying and combining genetic and clinical risk factors improves our understanding of HIV pathogenesis, provides epidemiologic information and has implications for treatment and prevention of neuropsychological impairment. For instance, from the genetic and virologic data acquired, the group with the lowest rate of neuropsychological decline (8%, which means virtually no decline given the use of the 90% confidence interval) would be: HLA DR*04 negative, at least one protective Class I allele, and HIV RNA undetectable. In contrast, the group with the highest rate of neurocognitive decline (45%) were HLA DR*04+ and detectable HIV RNA (a stronger influence in the DR*04 group than the presence or absence of a protective Class I allele).

The co-detection of contradictory markers underscores the difficulty of predicting neurocognitive decline and risk of HAND for any individual. A case in point is one Anhui participant who is homozygous for HLA DR*04, is ApoE4+, but also has 3 protective Class I alleles (B*27, A*03, A*33) and has plasma HIV RNA <2500 copies/ml, but has never been on ART. Her CD4 levels remain >500/mm^3^, but she was neuropsychologically impaired at baseline. At 12 months she tested neuropsychologically normal, without any CNS targeted treatment (her only medication was Penicillin) and was plasma HIV RNA undetectable.

The observation that no single gene unequivocally guarantees HAND suggests the interaction of multiple host genes and environmental factors come into play and influence neurocogntive status in the context of HIV infection. Although the significance levels for associations between HLA DR*04, neurocognitive impairment/decline and low levels of viremia were modest (p values around 0.04), the pattern was consistent throughout the analyses, regardless of the comparison groups. Given the diversity at the HLA locus, it is impressive that any single gene can be shown to have a significant impact on neurocognitive impairment or HIV RNA levels. The consistency of the DR*04 associations (high risk of HAND, lower HIV RNA) in the Anhui and US cohort are especially striking given differences between the cohorts with respect to some HLA alleles (DR*03, DR*09, B*48), gender percentages (40% female in Anhui versus 8% in US), HCV infection (90% in Anhui versus 30% in US), level of education (an average of 5 years in Anhui versus 12 in the US).

Mechanisms of many HLA associations have yet to be defined and much remains to be elucidated concerning interactions between alleles. Since individuals with HLA-DR*04 have demonstrated low CD4 responses to CMV, HSV, and the mitogen PHA in our previous studies, the low CD4 response appears to be non (antigen) specific, a result of low levels of DR*04 protein expression or poor interaction of DR*04 with peptides or signaling molecules [4]. Although there are always concerns that apparent HLA associations with any one allele are actually an association with a closely linked gene, the observation that the HLA Class I protective alleles and DR*04 sorted independently in our analyses (despite proximity on Chromosome 6), supports allele identification.

The extensive diversity at the HLA B locus (32 possible alleles for Anhui) makes single allele analysis difficult. In an attempt to simplify, a number of studies have examined HLA B locus data according to the expression of shared public specificities (Bw expression). This classification resolves the B alleles into B*w4, B*w6 and B*15. B*w4 is reportedly protective (regarding HIV disease progression) in US cohorts, but not in China [3], [6], [34]. We compared frequencies B*w4 and B*w6 in groups with neurocognitive impairment and decline in the Anhui cohort, but consistent with other China studies, no protective associations for B*w4 or detrimental effects of B*w6 were detected. HLA B*13 (has B*w4) was actually over-represented in the Anhui baseline impaired group, but this finding was not predicted and would not be significant with corrections. HLA B*15 was over-represented in the baseline impaired group and has been identified as a marker of disease progression in other Chinese HIV cohorts [3]. As noted in the results, a higher frequency of HLA B*46 was detected in the Anhui neuropsychological decliners. This allele is rare in non- Asians and was not part of our initial hypothesis, but other studies in China have reported associations with both HIV seroconversion and disease progression [2], [3]. The consistency of these HLA B associations will be assessed in future study populations.

Diversity among HIV strains and relative neurotropic character are important variables that were not addressed in this report. Preliminary results suggest that the Anhui plasma donor cohort is primarily infected with HIV-1 subtype B, with a few individuals positive for C.

It is possible that some of our correlates with baseline neurocognitive impairment are independent of HIV, reflecting mild autism spectrum disorder (DR*04) [22] or very early symptoms of Alzheimer’s disease (ApoE4) [32]. The association of HLA DR*04 with neurocognitive decline over a year with detectable HIV RNA, however suggests an HIV associated mechanism for that allele. On the other hand, consistent with other reports, the ApoE4 association with neurocognitive impairment in this study was not clearly linked to HIV disease (although ApoE4 has been associated with more rapid HIV disease progression) [35]. At present, there is insufficient longitudinal data to assess the effects of either ApoE4, HLA DR*04, or A*03, A*33, B*27, B*57, or B*58 on HIV disease progression in this cohort. The small number of HIV infected individuals with both HLA DR*04 and ApoE4 and lack of an HIV negative control group for HLA associations precludes further analysis. Our working hypothesis concerning the combinatorial effect on cognition is that the presence of the two alleles (ApoE4 and HLA-DR*04) in the same host predisposes to low levels of academic achievement (before HIV infection) leading to little cognitive “reserve” following HIV infection. Regardless of the role of HIV, it is certainly of interest that the co-localization of these two alleles in one host can have a profoundly negative impact on cognition.

Overall, the data presented support the initial hypothesis that HLA DR*04 has a negative impact on neurocognitive status and promotes neurocognitive decline in the context of active HIV disease, but is also associated with lower HIV plasma viremia, consistent with the idea of minimal CD4 T-cell activation. HLA Class I alleles that present conserved viral sequences are protective, but, as predicted by models of CD4/CD8 T-cell interdependence, the HLA Class I protective effect is minimized by the presence of HLA DR*04. In vitro modeling of T-cell activation as well as epidemiologic studies will be needed to confirm mechanisms.
